# Distinct Effects of Olanzapine Depot Treatment on Behavior and Muscarinic M1 Receptor Expression in the Triple‐Hit Wisket Rat Model of Schizophrenia

**DOI:** 10.1111/gbb.70015

**Published:** 2025-01-23

**Authors:** Gyongyi Horvath, Eszter Ducza, Leatitia Gabriella Adlan, Alexandra Büki, Gabriella Kekesi

**Affiliations:** ^1^ Department of Physiology Albert Szent‐Györgyi Medical School, University of Szeged Szeged Hungary; ^2^ Department of Pharmacodynamics and Biopharmacy, Faculty of Pharmacy University of Szeged Szeged Hungary

**Keywords:** cognition, M1 muscarinic receptor, multiple hit, olanzapine, schizophrenia

## Abstract

This study aimed to characterize the triple‐hit schizophrenia‐like model rats (Wisket) by the assessment of (1) behavioral parameters in different test conditions (reward‐based Ambitus test and HomeManner system) for a prolonged period, (2) cerebral muscarinic M1 receptor (M1R) expression, and (3) the effects of olanzapine treatment on these parameters. Wistar (control) and Wisket rats were injected for three consecutive weeks with olanzapine depot (100 mg/kg) and spent 4 weeks in large cages with environmental enrichment (HomeManner). The vehicle‐treated Wisket rats spent longer time awake with decreased grooming activity compared to controls, without changes in their active social behavior (sniffing, playing, fighting) obtained in HomeManner. Olanzapine treatment decreased most of these parameters, only the passive social interaction (huddling during sleeping) enhanced mostly in the Wisket rats on the injection day, which recovered within 4 days. In the Ambitus test, vehicle‐treated Wisket rats showed lower locomotor and exploratory activities and impaired cognition compared to control rats, deteriorating by olanzapine in both groups. In Wisket brain samples, the M1R mRNA expression was significantly lower in the cerebral cortex and elevated in the hippocampus, with no difference in the prefrontal cortex versus control. Olanzapine normalized the hippocampal M1R expression, but enhanced it in the prefrontal cortex. The triple‐hit Wisket model rats had impaired behavioral characteristics in both acute reward‐based test and undisturbed circumstances investigated for prolonged periods, and altered cerebral M1R expression. Chronic olanzapine treatment resulted deterioration of some parameters in control group, and could restore only few negative signs in model rats.

## Graphical abstract

The triple‐hit Wisket model rats had impaired behavioral characteristics in both acute reward‐based tests and undisturbed circumstances investigated for prolonged periods, and altered cerebral M1R expression. Chronic olanzapine treatment resulted deterioration of some parameters in control group, and could restore only few negative signs in this complex schizophrenia‐like rat model.

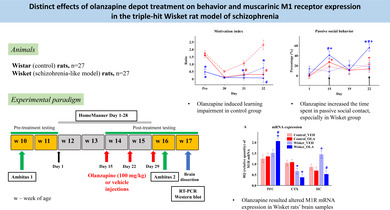

## 1 Introduction

Schizophrenia, as a neuropsychiatric disorder, is characterized by positive, negative, and cognitive symptoms with gene–environment interactions in its etiology. Therefore, to provide an animal model with high constructive validity for schizophrenia, a “multiple hit” rat substrain, termed Wisket, was developed from Wistar strain by combining environmental (postweaning social isolation), pharmacological (N‐methyl‐d‐aspartate [NMDA] receptor antagonist, ketamine, treatment) and genetic (selective breeding based on behavioral phenotype) manipulations [1, 2, 3]. Wisket rats exhibit a wide range of disturbances related to schizophrenia, including impaired sensory gating, cognitive function, and altered locomotor and exploratory activities [1, 3, 4, 5, 6]. Cognitive training improved these impairments and the antidiabetic metformin blunted clozapine induced side‐effects, supporting the predictive validity of this model [2, 7].

Multiple neurotransmitter systems, including the dopaminergic, glutamatergic and cholinergic ones, play role in the development of schizophrenia‐like symptoms [8]. Wisket rats exhibit changes in the expression and/or function of dopamine D1, D2, μ‐opioid, and oxytocin receptors [4, 9, 10, 11]. Cholinergic cells of the brain, located in the magnocellular basal forebrain, send inputs to different cortical and subcortical areas to activate muscarinic and nicotinic receptors [12, 13]. The muscarinic cholinergic system includes five G‐protein coupled receptor subtypes (M1R–M5R) [14]. Muscarinic M1R is abundant in the cerebral cortex (CTX), prefrontal cortex (PFC), and hippocampus (HC), playing significant roles in a range of brain functions, including behavioral activities and cognitive ability [14, 15]. Because these functions are affected in schizophrenia, the M1R‐mediated neurotransmission may play an important role in the etiology and treatment of schizophrenia. Accordingly, reduced levels of central M1R have been observed in schizophrenic patients; however, other studies could not confirm these data [16, 17, 18]. Furthermore, the activation of M1R produced antipsychotic‐like effects and/or cognitive enhancement, due to the modulation of the functional connections in the brain [19, 20].

Although positive symptoms are adequately addressed by antipsychotic treatments, they remain largely ineffective for negative and cognitive signs, or at least the results are inconsistent, which is also true for the frequently applied atypical antipsychotic drug, olanzapine [21, 22]. The effects of the antipsychotics on the motor activity, cognition, social behavior, or sensory gating have been controversial, probably due to the differences between the patients' characteristics or the applied animal models and/or the behavioral tests [23, 24, 25, 26]. Olanzapine acts on multiple receptors, including dopaminergic, serotonergic, and muscarinic receptors [27]. It has high affinity for M1R, which can influence M1R expression [28]. Several types of single hit schizophrenia models have revealed that olanzapine treatment decreased the learning impairment in various behavioral tests [29, 30, 31, 32, 33, 34, 35]. However, other studies found that olanzapine disrupted the cognitive function in control animals, or did not improve the impairments in schizophrenia‐like animals, primarily due to its sedative effects [36, 37, 38, 39, 40, 41, 42, 43, 44, 45, 46].

This study aimed to characterize the triple‐hit Wisket rat model of schizophrenia by the assessment of (1) the behavioral parameters in acute test (Ambitus) and under prolonged observation (4 weeks; HomeManner system; large cage), (2) the cerebral muscarinic M1 receptor (M1R) expression, and (3) the effects of olanzapine treatment on these parameters.

## 2 Materials and Methods

### 2.1 Animals

Control (Wistar, *n* = 27) and Wisket (*n* = 27) male rats were maintained under a 12 h light/dark cycle and controlled temperature (22°C ± 1°C). Before the reward‐based cognitive test was performed (Ambitus 1 and 2, see below Section 2.2), the rats were food‐deprived for 2 days, but water was freely available. Moderate food restriction was also maintained throughout the posttreatment Ambitus tests (for 3 days) with a decreased amount of food (10–15 g/day). The body weight of rats was controlled during the whole experiment. The behavioral tests were performed between 08:00 a.m. and 4:00 p.m. The Hungarian Ethical Committee for Animal Research (registration number: XIV/1248/2018) approved all experiments, which were carried out in accordance with the guidelines set by the Government of Hungary and EU Directive 2010/63EU for animal experiments.

### 2.2 Ambitus Test

**FIGURE 1:**
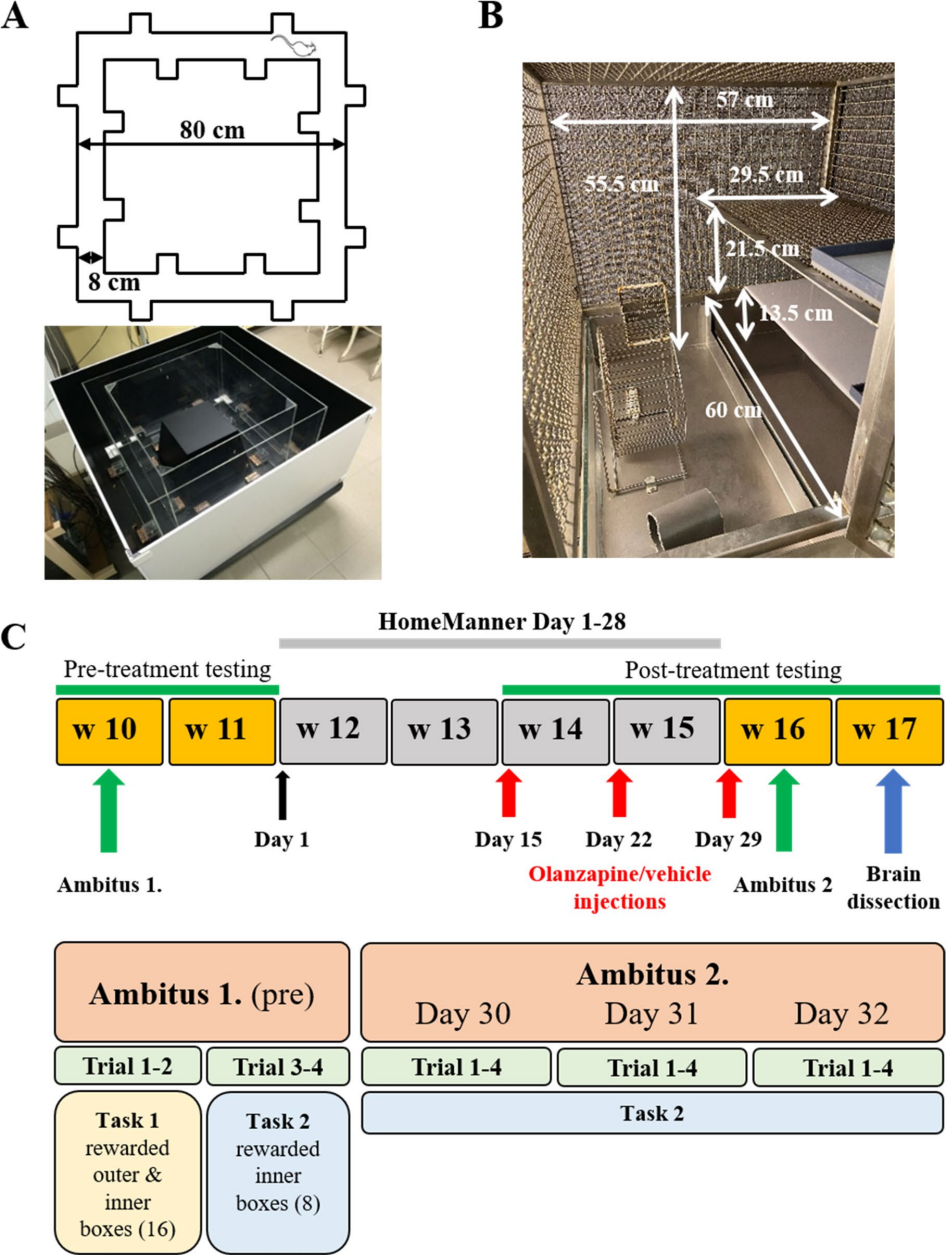
The structure of the Ambitus (A) and the HomeManner system (B) and the experimental paradigm (C), indicating the timeline of behavioral and pharmacological interventions. W: week.

The Ambitus apparatus, used for reward‐based cognitive testing, is a rectangular corridor constructed of clear Plexiglas on a black floor (Figure 1A; www.deakdelta.hu) [1]. As we described in detail earlier, the Ambitus system is the combination of the reward‐based Hole Board and maze (corridor) tests, which is suitable to detect the locomotion, exploratory activity and the motivation for eating rewards (as a sign of cognitive function) in a simple rectangular corridor [1]. Therefore, no extra navigation ability is required for task completion, and both the negative and cognitive impairments can be reliably detected in our triple hit model [1]. Each corridor has four side boxes (at the internal and external sides of the corridor, altogether 16) for food rewards (puffed rice: 20 mg). Infrared beams detect the exploratory activity and the collection of the rewards at each side box and the locomotor activity in the midway of each corridor with 1 ms time resolution. After the food rewards (puffed rice) were inserted, the trials were commenced by placing the rats at the starting point (Figure 1A). The experimenter left the room immediately, and the rats were allowed to explore the corridor and collect food rewards for 300 s. The apparatus was cleaned with 70% alcohol between animals.

### 2.3 HomeManner System

The HomeManner (HM) system comprised six sets of operant cages (Figure 1B) in a separate room with house‐light outside the cages, under standard conditions, as described [47]. Each cage had three levels, the sidewalls and the top of the cages were made of steel wire grid. Because a structured cage environment may be more beneficial for rodents, than a large floor area [48], the cage was divided into two parts with one and three floors, respectively. The rats could reach all the floors by climbing on the grid wall. In the single‐story part, a playing area was equipped with an abacus, and a plastic tube for environmental enrichment. On its front side a drinking bottle was provided for free access to tap water. The first level of the three‐floor side was provided as a shelter (28.5 × 60 × 11 cm) with bedding material (3–4 cm thick sawdust), in which the rats also could bury marbles. The second and third levels were above the shelter and made from opaque plastic and steel grids, respectively. Food was freely available in a tray on the second floor. The experiments were recorded using an infrared video device (WCM‐21VF, CNB, China).

### 2.4 In Vitro Experiments

After the behavioral assessment was completed, the rats were terminated, their brain removed and the following areas were dissected immediately on dry ice: hippocampus (HC); prefrontal cortex (PFC; an approximately 1.5 mm coronal slice was hand‐dissected to prevent the white matter from dissection), and the rest of the cortex (CTX) [49]. The tissues were frozen in liquid nitrogen and stored at −75°C until in vitro assays.

#### 2.4.1 Total RNA Preparation

Total cellular RNA was isolated as described [50], by extraction with guanidinium thiocyanate‐acid‐phenol‐chloroform, precipitated with isopropanol, the RNA was washed with 75% ethanol, and resuspended in diethyl pyrocarbonate‐treated water. RNA purity was controlled at an optical density of 260/280 nm with BioSpec Nano (Shimadzu, Japan). All samples exhibited an absorbance ratio in the range of 1.6–2.0. RNA quality and integrity were assessed by agarose gel electrophoresis.

#### 2.4.2 Real‐Time Quantitative Reverse‐Transcriptase PCR

Reverse transcription and amplification of the PCR products were performed by using the TaqMan RNA‐to‐*C*_T_‐Step One Kit (Thermo Fisher Scientific, Hungary) and an ABI StepOne Real‐Time cycler. Reverse‐transcriptase PCR amplifications were performed as follows: 48°C for 15 min and 95°C for 10 min, followed by 40 cycles at 95°C for 15 s and 60°C for 1 min. The generation of specific PCR products was confirmed by melting curve analysis. The following primers were used: assay ID Rn00589936_s1 for M1R and Rn00667869_m1 for β‐actin as endogenous control (Thermo Fisher Scientific, Hungary). All samples were run in triplicate. The fluorescence intensities of the probes were plotted against the cycle number of PCR. The amplification cycle displaying the first significant increase of fluorescence signal was defined as the threshold cycle (*C*_T_).

#### 2.4.3 Western Blot Analysis

Brain tissues were homogenized in RIPA Lysis Buffer System, containing phenylmethylsulfonyl fluoride, sodium orthovanadate, and a protease inhibitor cocktail, using a Micro‐Dismembrator (Sartorius AG, Germany) and centrifuged at 11.000× *g* for 30 min at 4°C. Total protein amounts in the supernatant were determined by spectrophotometry (BioSpec‐nano, Shimadzu, Japan). Then, 25 μg of sample protein per well was subjected to electrophoresis on 4%–12% NuPAGEBis‐Tris Gel in XCellSureLock Mini‐Cell Units (Thermo Fisher Scientific, Hungary). Proteins were transferred from gels to nitrocellulose membranes using iBlot Gel Transfer System (Thermo Fisher Scientific, Hungary). The Ponceau S (Sigma‐Aldrich, Hungary) was used to check the standard running and transfer conditions. The blots were incubated overnight on a shaker with M1R (55 kDa) and β‐actin (42 kDa) polyclonal antibodies (Thermo Fisher Scientific, Hungary; diluted 1:200) in blocking buffer. Antibody binding was detected with the WesternBreeze Chromogenic immunodetection kit (Thermo Fisher Scientific, Hungary). Images were captured with the EDAS290 imaging system (Csertex Ltd., Hungary), and the optical density of each immunoreactive band was determined with Kodak 1D Images analysis software. Optical densities were calculated as arbitrary units after subtraction of the local area's background. All samples were run in triplicate.

### 2.5 Experimental Protocol

For pretreatment behavioral assessment rats were involved in the Ambitus test 1 at the age of 10 weeks, where two different tasks were applied: in Task 1 (trials 1 and 2) all the internal and external boxes were baited (16 rewards), and in Task 2 (trials 3 and 4) only the internal boxes were baited (8 rewards) (Figure 1C). The rats performed two sessions (two trials/session, about 2 min apart) one in the morning and another 3 h later. At the age of 12 weeks, the animals were transferred to the HM system for 4 weeks (started on Day 1), with three rats/cage. Following 2‐weeks of habituation, the animals were treated with olanzapine pamoate depot formulation (Zypadhera, Eli Lilly Nederland B.V.; 100 mg/kg) or its vehicle solution (640 μL/kg) administered intramuscularly on three consecutive weeks (on Days 15, 22, and 29 at 9:00 a.m.), as described [51]. The animals were randomly assigned into four groups: control‐vehicle (Control_VEH; *n* = 12), control‐olanzapine (Control_OLA; *n* = 15), Wisket‐vehicle (Wisket_VEH; *n* = 12), and Wisket‐olanzapine (Wisket_OLA *n* = 15). Rats' behavior in the HM system was video recorded, and the behavioral patterns were analyzed offline for two 1 h periods (between 18:00–19:00 and 19:00–20:00 p.m., at the beginning of the dark phase) on Day 1, on the treatment days (Days 15 and 22) to observe the acute effects of olanzapine; and 4 days after the first injection (on Day 19) to detect the prolonged effects of olanzapine. Since only the data from the first 2 h of the dark phase were analyzed, we cannot draw any conclusion about the circadian rhythm of the animals. The time spent awake, grooming, and in active (sniffing, following, playing, and fighting), and passive social (huddling during sleeping) interactions were detected by an expert technician. The awakening time was calculated as the total time minus sleeping time. Sleeping was defined as a perfect inactivity in sleeping position [52]. Since the amount of the activities depend on the duration of awake (grooming, active social behavior) or sleeping (passive social behavior) phases, the raw behavioral data were transformed to their percentage for statistical analysis. After the third injection (on Day 29; 16 weeks of age), the animals were transferred to standard cages (Eurostandard Type III) and were involved in a 3‐day‐long Ambitus test 2 on Days 30–32 (Ambitus 2; Figure 1C) with Task 2 (only the internal boxes were baited: 8 rewards). The Ambitus system is suitable to detect and/or calculate several parameters, including locomotor, exploratory and reward collecting (eating) activities, and cognition related ones (effective exploration, motivation index), as their definitions and calculations are shown in Table 1. At the age of 17 weeks 6–6 rats/group were randomly selected and terminated for in vitro study sampling.

**TABLE 1:** Behavioral outcomes.

System	Parameter	Unit	Definition and calculation
Ambitus	Locomotor activity (locomotion)	N	The number of entries into the corridors of the Ambitus apparatus within a 5‐min trial. Direct count of corridor entries.
	Exploration_Total (total exploration)	N	The total number of visits to the side boxes (both internal and external) in the Ambitus system during the 5‐min trial. Direct count of visits to side boxes.
	Exploration_Before_External (external exploration)	N/300 s	The number of visits to external boxes normalized by the time to collect all rewards (expressed per 300 s). [Number of external box visits/Time to collect all rewards] × 300.
	Exploration_Before_Internal (internal exploration)	N/300 s	The number of visits to internal boxes normalized by the time to collect all rewards (expressed per 300 s). [Number of internal box visits/time to collect all rewards] × 300.
	Motivation index	Ratio	A ratio indicating the animal's motivation to collect rewards. [Number of collected rewards × 300/(maximum number of rewards × task completion time (s))].
	Effective exploration ratio	Ratio	The ratio of the number of collected rewards to the number of reward‐containing boxes explored. (Number of collected rewards)/(number of reward‐containing boxes explored).
HomeManner	Time spent awake (awake %)	%	The percentage of time during observation when the animal is awake (calculated as total observation time minus sleeping time). [(Observation time − sleep time)/observation time] × 100.
	Grooming behavior (grooming %)	%	The percentage of awake time spent grooming. (Time spent grooming/Awake time) × 100.
	Active social behavior (active social %)	%	The percentage of awake time spent engaging in social interactions such as sniffing, following, playing, or fighting. (Time spent in active social behavior/awake time) × 100%.
	Passive social behavior (passive social %)	%	The percentage of sleeping time spent in passive social contact (huddling together during sleep). (Time spent in passive social contact/sleep time) × 100%.

### 2.6 Behavioral Outcomes

The following behavioral parameters were analyzed to assess the animals' exploratory behavior, reward‐related motivation, and social engagement. The parameters were grouped into two categories based on the experimental systems used: Ambitus Test Parameters and HomeManner System Parameters.

In the Ambitus system, locomotor activity, defined as the number of entries into the corridors of the apparatus during a 5‐min trial, was used as a measure of general movement and exploratory drive. This parameter was calculated by directly counting the number of corridor entries made by the animals. Total exploration captured the overall exploratory behavior by recording the total number of visits to both internal and external side boxes within the Ambitus system during the same trial period.

To differentiate between specific types of exploration, external exploration was assessed by counting visits to external boxes before the animals collected all available rewards. The number of visits was normalized to the time taken to collect the rewards and expressed per 300 s (cut‐off time). Similarly, internal exploration was defined as the number of visits to internal boxes during the same period, normalized in the same way to reflect exploration prior to task completion. These measures allowed a more detailed assessment of how the animals distributed their exploratory activity in relation to the external versus internal side of the corridor.

The motivation index provided insight into the animals' eagerness and ability to complete the reward‐based task efficiently. It was expressed as a percentage and calculated using the formula: (number of collected rewards × 300)/(maximum number of rewards × task completion time in seconds). This index reflected the efficiency with which the animals approached the task and their drive to collect rewards. Additionally, the effective exploration ratio measured the focus of exploration by calculating the ratio of the number of collected rewards to the number of reward‐containing boxes explored (8 for task 1 and 16 for task 2). This parameter reflected the effectiveness of the animals' exploratory behavior in obtaining rewards.

In contrast, the HomeManner system parameters focused on sleep–wake cycles, grooming, and social interactions in undisturbed conditions. Time spent awake was defined as the percentage of total observation time during which the animals were awake, calculated by subtracting the time spent sleeping from the total observation time and expressing the result as a percentage. Sleeping was defined as a perfect inactivity in sleeping position [52]. Grooming behavior was assessed as a measure of self‐care and activity levels, expressed as the percentage of awake time spent grooming.

Social behaviors were divided into active and passive categories. Active social behavior was defined as the percentage of awake time the animals spent engaging in social interactions, such as sniffing, following, playing, or fighting. In contrast, passive social behavior was measured as the percentage of sleeping time during which the animals engaged in passive social contact, such as huddling together with conspecifics during rest. These measures provided insight into both the active engagement and social bonding tendencies of the animals during rest.

By combining data from the Ambitus and HomeManner systems, these behavioral parameters allowed for a comprehensive analysis of movement, exploration, motivation, and social interaction across different contexts. The above parameters are summarized in Table 1.

### 2.7 Statistical Analysis

Repeated measures of ANOVA was applied for all in vivo parameters by group and treatment, including the weekly registered body weight, the fluid‐ and food consumption in the HM (as pre vs. posttreatment sets), the Ambitus test related parameters (the mean of the four trials/day) and the pooled behavioral data for 2 h in the HM, because no significant differences were observed between the two 1 h period (obtained on Days 1, 15, 19, and 22) in the analyzed parameters. Post hoc comparisons were performed by using the Fisher LSD test. Factorial ANOVA was used for the in vitro data with the group and treatment as factors. All data are expressed as means ± SEM, and significance was accepted at the *p* < 0.05 level. For the statistical analysis, Statistica 13.4.0.14 (TIBCO Software Inc., USA) software was used.

## 3 Results

### 3.1 Basal Parameters

Regarding the body weight, significant effects of group, time, group, and time, and treatment and time interactions were observed (Table 2). Wisket rats had lower body weight during the pretreatment period (weeks 9–11) compared with controls (260 ± 6.9 vs. 310 ± 8.6 g, respectively, at the age of 10 weeks). While the Wisket_VEH group reached the controls' weight during the HomeManner experiments, the Wisket_OLA group had significantly lower body weight than its matched control during the treatment phase (338 ± 16.0 vs. 391 ± 12.4 g, respectively, at the age of week 17).

**TABLE 2:** Results of basal parameters analyzed repeated measurements (*F* value; [degree of freedom] *p* value).

Parameters	Group (GR)	Treatment (TR)	Time (T)	GR/T	TR/T
Body weight	13.25; (1,50) < 0.001		358.67; (7,350) < 0.0001	5.11; (7,350) < 0.0001	7.01; (7,350) < 0.0001
Fluid consumption	7.20; (1,68) < 0.01	6.84; (1,68) < 0.05			
Food consumption	9.97; (1,68) < 0.005	4.01; (1,68) < 0.05			4.33; (1,68) < 0.05

Significant effects of group and treatment were found in both fluid and food consumptions (Table 2), and the post hoc analyses disclosed significant differences in the postinjection fluid and food intakes; that is, the Wisket_VEH group had increased fluid (63.1 ± 8.70 vs. 47.8 ± 4.68 mL/kg/day), and food (27.1 ± 0.76 vs. 22.4 ± 0.76 g/kg/day, respectively) intakes compared with Wistar_VEH animals (Table 2). Furthermore, olanzapine treatment significantly enhanced the food intake in the control animals (Control_OLA: 26.4 ± 1.05 vs. Control_VEH: 22.4 ± 0.81 g/kg/day, respectively).

### 3.2 Olanzapine Treatment Induced Behavioral Alterations

#### 3.2.1 Ambitus Test

**FIGURE 2:**
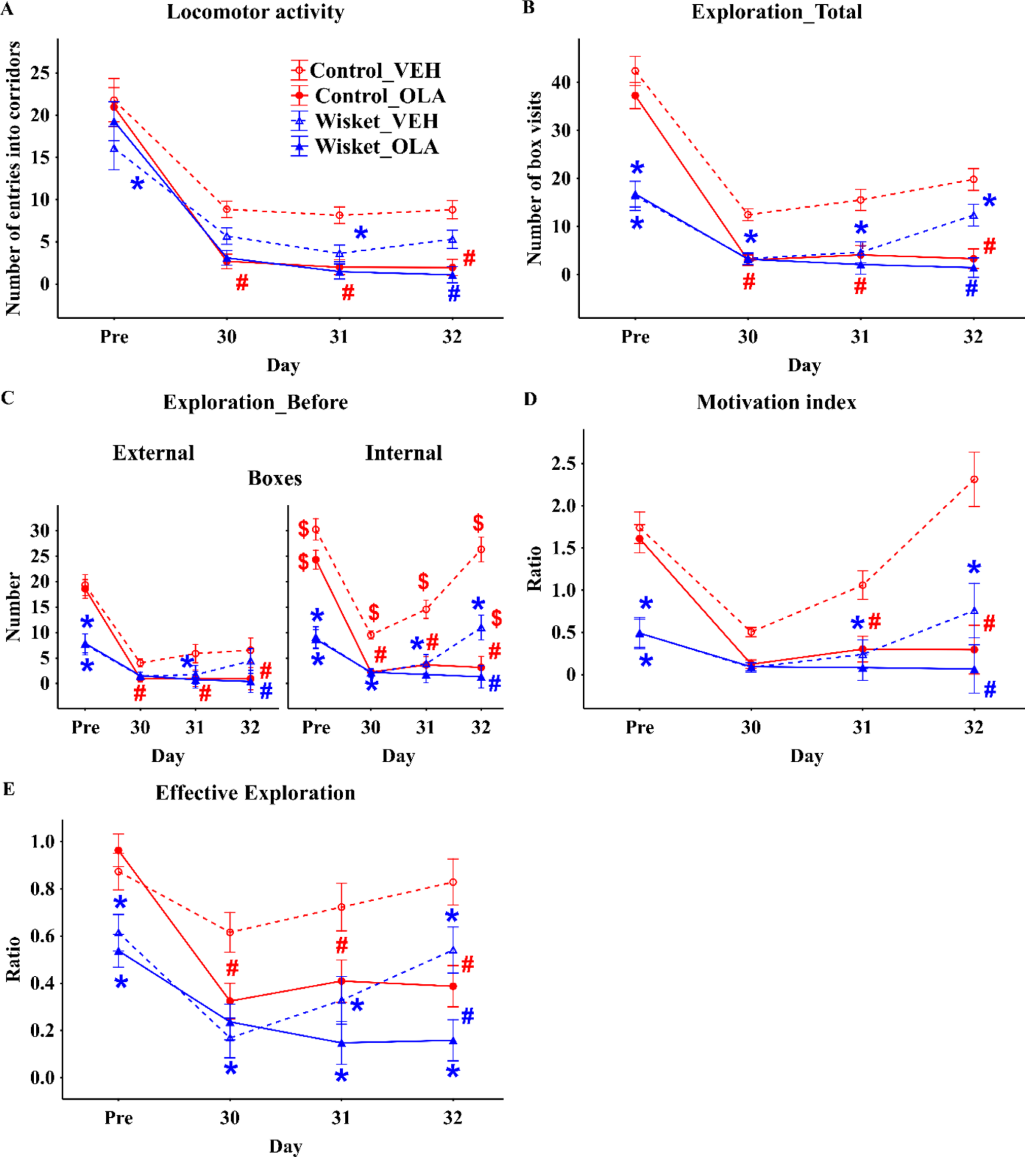
(A–E) Behavioral characterization of the different groups in the Ambitus test. Data are presented as means ± SEM. Symbols denote significant (*p* < 0.05) differences by group (*) and drug treatment (#), and external side ($). Definitions and calculations of the parameters are presented in the Table 1.

In agreement with our earlier studies, the vehicle‐treated Wisket animals had several behavioral alterations in the hole‐board, the novel object recognition, and the Ambitus test compared with controls [1, 2, 6] (Figure 2A–E). Thus, Wisket group had lower locomotion and exploration during the whole investigated period compared to control animals (Figure 2A,B). Separate analysis of the exploration to the external and internal boxes showed significantly lower activities in the Wisket_VEH group compared with the matched controls, primarily to the internal boxes (Figure 2C). Furthermore, significantly higher exploration frequency could be detected in the internal boxes in Control_VEH group during the whole period compared to external ones, while this phenomenon appeared only on Day 32 in the matched Wisket_VEH group (Figure 2C). The cognition‐related parameters (motivation index and effective exploration) were also disturbed in the model animals during the whole period (Figure 2D,E). Significantly lower behavioral activity and motivation index value was observed on the first day of the Ambitus test 2 (at 16 weeks old) compared with data obtained in the Ambitus test 1 (at 10 weeks old) in the vehicle‐treated control animals, but most of the parameters improved gradually in both groups during the 3 days, with lower steepness in the Wisket animals (Figure 2A–E).

All the investigated parameters were significantly impaired by olanzapine treatment in the control animals in Ambitus test 2. Similar phenomenon was detected in the Wisket group, however, probably due to the floor effects, this phenomenon was significant only at Day 32. General behavioral improvement and better task performance in vehicle‐treated groups during the 3 days of Ambitus test 2 was observed, but it did not appear in olanzapine‐treated groups. Therefore, no significant differences were observed between the olanzapine‐treated groups in most of the parameters (Figure 2A–E). Separate analysis of the exploration to the external and internal boxes also showed that olanzapine treatment resulted high level of inactivity in both groups, furthermore, the no significant side differences could be detected between olanzapine‐treated groups (Figure 2C). However, between the Wisket and control animals, the significant differences in the effective exploration persisted during the olanzapine treatment (Figure 2E).

#### 3.2.2 HM System

**FIGURE 3:**
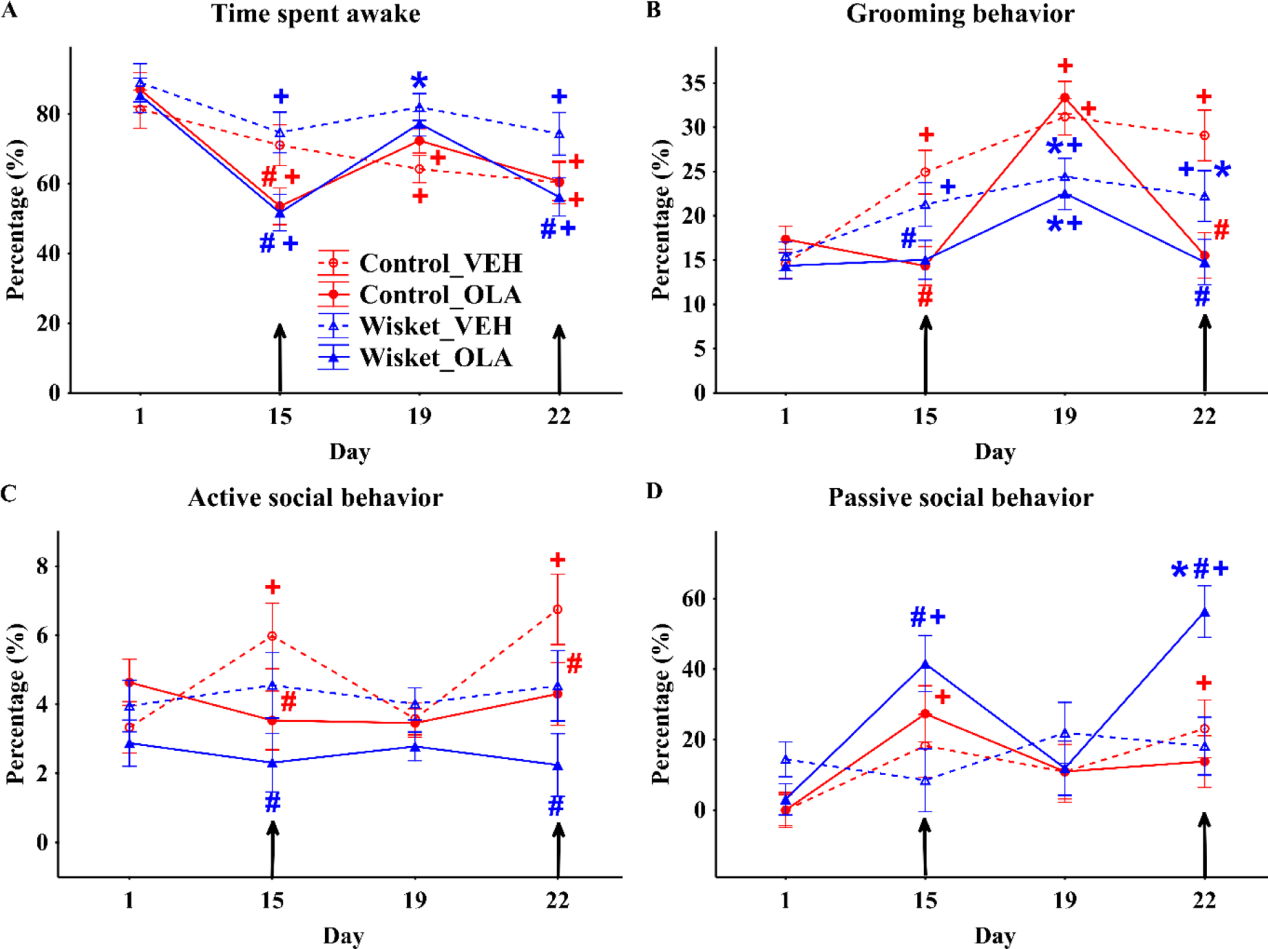
Behavioral characterization of the different groups in the HM system. Definitions and calculations of the parameters are presented in the Table 2. The arrow indicates injection day. Symbols denote significant (*p* < 0.05) differences by group (*) and drug treatment (#), and compared with pretreatment day (+). Definitions and calculations of the parameters are presented in the Table 1.

Significantly longer time awake was observed in the vehicle‐treated Wisket group compared with its matched control (Table 3 and Figure 3A). Both Wistar and Wisket rats spent shorter time awake on the days of olanzapine injections compared with Day 1 and to the vehicle‐treated animals, which recovered within 4 days. A continuous increase in the grooming time was observed in both vehicle‐treated groups during the whole investigation period; however; it was significantly shorter in the Wisket compared with the control animals (Table 3, Figure 3B). Olanzapine treatment significantly decreased the time spent grooming in both groups compared to their vehicle‐treated counterparts on the treatment days, which recovered within 4 days. The active social behavior revealed significant effects of group, treatment, and treatment and time interaction (Table 3 and Figure 3C). Post hoc analysis showed that Control_VEH rats spent longer time with active social contacts on the day of vehicle injection than on noninjection days, which phenomenon was not observed in the Wisket_VEH group, that is, they showed continuously low level of active social contacts. Olanzapine treatment did not influence significantly the duration of active social contacts in either the control or Wisket group compared with Day 1. Regarding the duration of passive social contact (huddling during sleeping), while no significant differences could be detected between the vehicle‐treated groups, the Wisket_OLA group spent significantly longer time in passive social contact on the days of olanzapine injection compared with the Wisket_VEH group and to Day 1 (Figure 3D).

**TABLE 3:** Results of the behavioral analyses (*F* value; (degree of freedom) *p* value).

Parameter	Group (GR)	Treatment (TR)	Time (T)	GR/TR	GR/T	TR/T	GR/TR/T
Ambitus							
Locomototion	8.20; (1,50) < 0.01	14.26; (1,50) < 0.0005	118.80; (3,150) < 0.0001	4.35; (1,50) < 0.05		4.62; (3,150) < 0.005	
Total exploration	37.99; (1,50) < 0.0001	19.56; (1,50) < 0.0001	157.74; (3,150) < 0.0001	5.41; (1,50) < 0.05	28.53; (3,150) < 0.0001	8.23; (3,150) < 0.0001	
External exploration	35.23; (1,50) < 0.0001	12.87; (1,50) < 0.0001	153.26; (3,150) < 0.0001		30.35; (3,150) < 0.0001	4.84; (3,150) < 0.005	
Internal exploration	30.59; (1,50) < 0.0001	19.59; (1,50) < 0.0001	46.58; (3,150) < 0.0001	6.98; (1,50) < 0.05	12.24; (3,150) < 0.0001	11.43; (3,150) < 0.0001	
Motivation index	26.15; (1,50) < 0.0001	14.03; (1,50) < 0.0005	25.17; (3,150) < 0.0001	4.90; (1,50) < 0.05	6.88; (3,150) < 0.0005	13.25; (3,150) < 0.0001	
Effective exploration ratio	19.49; (1,50) < 0.0001	7.94; (1,50) < 0.01	34.89; (3,150) < 0.0001			8.71; (3,150) < 0.0001	3.14; (3,150) < 0.05
HomeManner							
Time spent awake		4.16; (1,32) < 0.05	22.77; (3,96) < 0.0001			5.08; (3,96) < 0.005	
Grooming behavior	6.94; (1,32) < 0.05	9.80; (1,32) < 0.005	30.58; (3,96) < 0.0001		3.48; (3,96) < 0.05	9.42; (3,96) < 0.0001	
Active social behavior	5.08; (1,32) < 0.05	8.18; (1,32) < 0.01				2.91; (3,96) < 0.05	
Passive social behavior	4.67; (1,32) < 0.05		8.73; (3,96) < 0.0001			3.64; (3,96) < 0.05	3.97; (3,96) < 0.05

*Note:* For a detailed description of the outcome parameters, see Section 2.6 and Table 1.

The results of the behavioral analyses are summarized in Table 3.

### 3.3 In Vitro Results

**FIGURE 4:**
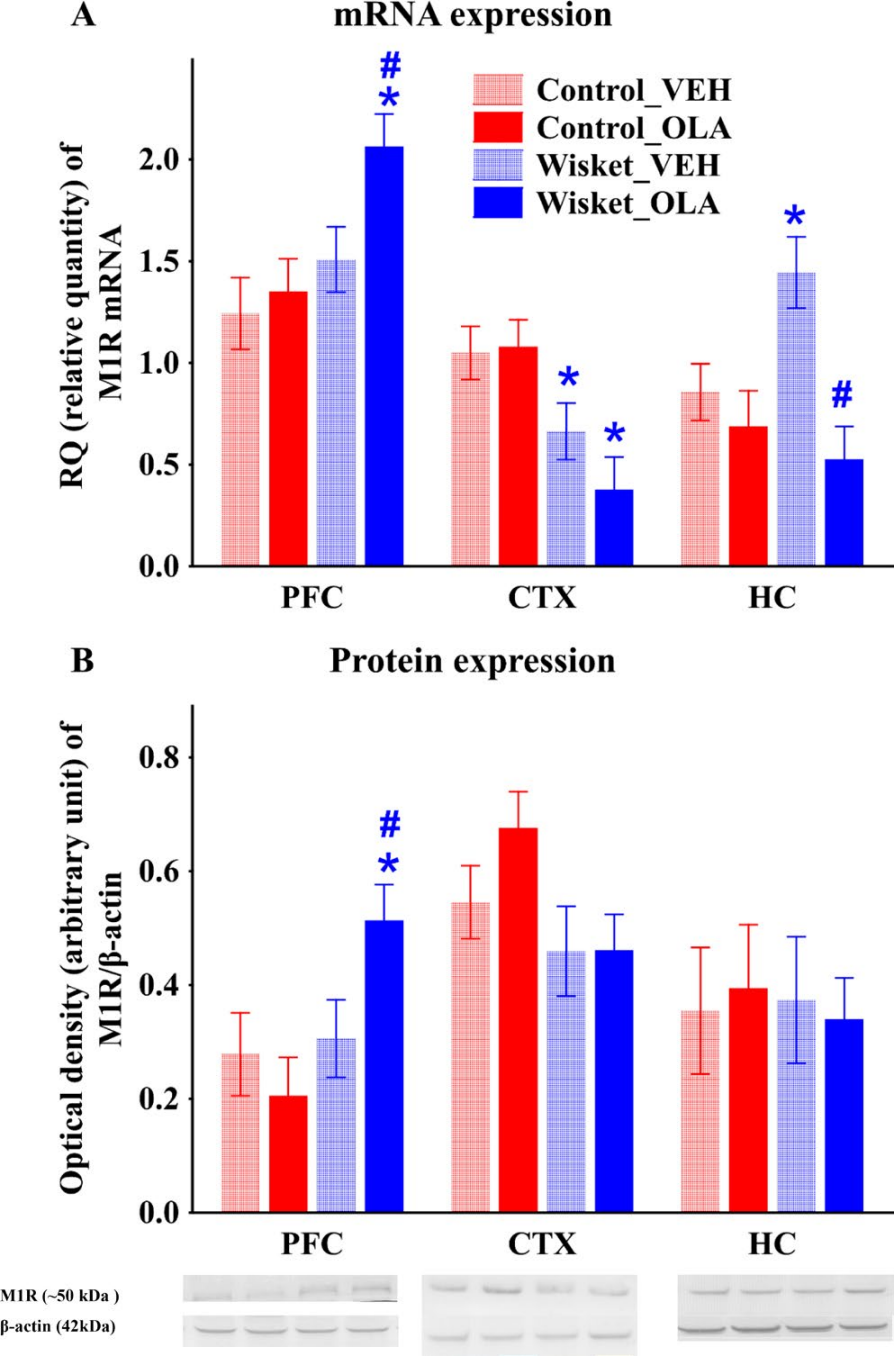
Results of RT‐PCR and Western immunoblotting experiments. The changes of mRNA and protein expressions of M1R in prefrontal cortex (PFC), cortex (CTX), and hippocampus (HC). Data are presented as means ± SEM. Symbols denote significant (*p* < 0.05) differences by group (*) and drug treatment (#).

Regarding the M1R mRNA and protein expressions in the PFC, factorial ANOVA showed significant effects of group (8.50; [1,19]; *p* < 0.01; 27.00; [1,27]; *p* < 0.0001; respectively), furthermore, the treatment (4.30; [1,27]; *p* < 0.05, respectively), and group and treatment interaction (18.88; [1,27]; *p* < 0.0005) was also significant for the protein expression. Thus, while no significant differences were between the vehicle‐treated group samples, significantly enhanced M1 mRNA and protein expressions were observed in the Wisket_OLA group compared with both the Control_OLA and the Wisket_VEH samples (Figure 4A,B). The M1R mRNA expression of in the CTX showed significant effects of group (15.60; [1,28]; *p* < 0.0005), thus, significantly lower expression was measured in the Wisket animals compared with controls in both the vehicle and olanzapine treated groups, and this difference slightly enhanced by olanzapine treatment. While the tendency in the M1R protein expression was similar to the mRNA expression, the changes in the Wisket groups did not reach significant effects. Significant effects of treatment (10.83; [1,20]; *p* < 0.005) and group and treatment interaction (5.14; [1,20]; *p* < 0.05) were observed in the HC, with significantly higher expression of M1R mRNA in the Wisket_VEH samples compared with controls, which was significantly decreased by olanzapine treatment. No significant effects of treatment and/or group could be detected in the M1R protein expression (Figure 4B).

## 4 Discussion

This study revealed that the triple‐hit schizophrenia‐like model rats show behavioral impairments not only in an acute reward‐based test but in undisturbed circumstances during a prolonged observation period as well. Additionally, they had altered, region specific cerebral M1R mRNA, but not protein expression. Repeated olanzapine depot administration reduced behavioral activities in both groups either in the acute reward‐based test or in the HM system, but it enhanced the passive social behavior and the M1R expression in the PFC in the Wisket animals.

Clinical and preclinical data regarding the effects of antipsychotic treatments are inconsistent, probably due to the differences between patients characteristics, applied animal models, and/or behavioral tests [23, 24, 25, 26]. Several long‐acting parenteral antipsychotic drugs are available in clinical practice, including olanzapine pamoate. Single intramuscular injection to patients given at intervals of 1–4 weeks produces adequate plasma concentrations; furthermore, they have fewer neurological adverse effects and are better tolerated [53]. Although the pharmacokinetics, clinical actions and metabolic effects of olanzapine depot have already been studied [51, 54, 55, 56], this is the first on its behavioral effects in preclinical settings.

The negative signs and cognitive deficit in schizophrenia are associated with poor functional outcomes that may be further aggravated by antipsychotic treatment. In agreement with earlier preclinical studies, olanzapine treatment significantly decreased the locomotor activity in both groups of animals, with a lower effect in the Wisket group, probably due to the floor effect [31, 57, 58]. Our results showed that besides few beneficial effects of olanzapine, it caused acute sedative effects, which recovered (at least partially) within a few days. These results are in agreement with the results of other studies where single or repeated olanzapine treatments were applied [21, 22].

Several types of single hit schizophrenia models detected that olanzapine treatment, at least partially, reversed schizophrenia‐related learning impairments in different behavioral tests [29, 30, 31, 32, 33, 34, 35, 59, 60]. However, other studies, in agreement with our results, showed that olanzapine did not improve these impairments in schizophrenia‐like rodents, or even it disrupted cognitive function in control animals, which might be related, at least partially, to its prolonged sedative effects (for 3 days in the Ambitus test) [26, 36, 37, 38, 39, 40, 41, 42, 43, 44, 45, 46].

Although, the time spent awake during the investigated hours did not show significant difference between the vehicle‐treated groups on Days 1 and 15 in the HM, its continuous decrease in the Wisket_VEH group led to enhanced differences between the two groups afterwards, indicating impaired sleep–wake rhythm. While this paradigm was not appropriate for the detailed investigation of the daily activity of these animals, impaired circadian rhythm, as a well‐known characteristic of schizophrenic patients and animal models of schizophrenia, was also proved in the Wisket rats in our earlier studies [5, 61, 62, 63, 64]. Olanzapine depot treatment decreased the time spent awake in both groups on the days of injections, which recovered within 4 days, indicating the sedative effect of olanzapine administration [65].

Deficits in personal care are frequently observed in schizophrenic patients [66]. Rodent self‐grooming, beside its high importance in hygienic behavior, is also related to general activity, mostly investigated in acute circumstances [67, 68]. Its prolonged observation in the HM system showed that grooming activity was lower in the Wisket rats, in accordance with the human phenomenon. Furthermore, olanzapine acutely decreased this behavior on the day of its injection in both groups, suggesting again the high level of inactivity in both groups.

Impairments in social interactions are well known in schizophrenic patients and in animal models of schizophrenia, too [31, 69, 70, 71, 72, 73, 74]. Despite the impaired social activity with increased aggression and active social withdrawal in Wisket rats investigated in acute social test [70], this phenomenon was not observed in Wisket‐VEH group under undisturbed circumstances for the prolonged investigation period. Surprisingly, vehicle injection (as an important stress factor) significantly enhanced the active social behavior of the control animals even about 8 h after the injection. This response was not observed in the Wisket rats, suggesting their blunted sensitivity to this intervention. The findings according to the effect of olanzapine on social deficit are controversial in preclinical studies, that is, some studies have reported no effects [38, 40, 57], while others have found improvements [31, 69], applying acute social interaction tests. However, both olanzapine treated groups spent significantly less time in active social interaction compared with their vehicle treated matched groups on the day of injection in undisturbed conditions, suggesting again its sedative properties. By contrast, olanzapine treatment substantially increased the huddling (passive social) behavior primarily in the Wisket animals. Since huddling during sleeping is a motivated behavior [75], it can be suggested as a beneficial effect of olanzapine treatment in Wisket animals.

Besides the widely accepted dopaminergic and glutamatergic hypothesis of schizophrenia, the involvement of cholinergic receptors in its etiology is also important [8, 76]. M1Rs are widely expressed in the brain predominantly postsynaptically on dopaminergic neurons and colocalized with NMDA receptors [15, 77], therefore, their dysfunction in schizophrenia may contribute to the symptoms through the modulation of the dopaminergic and/or glutamatergic system [12, 14, 24, 77, 78, 79, 80]. M1R activation produced antipsychotic‐like effects and/or cognitive enhancement, which might have been due to the modulation of the functional connectivities in the brain [14, 19, 20, 81, 82]. Reduced cholinergic signaling and decreased M1R expression in the CTX and PFC, but not in the HC were reported in schizophrenic patients [12, 17, 79, 83, 84]. Similarly, in a single hit animal model of schizophrenia also decreased expression of M1R mRNA in the CTX was observed, which was enhanced by antipsychotic treatment [85]. It is well‐known that olanzapine has marked affinity for M1R with antagonistic effects [14], so it may further compromise cognitive functions. However, olanzapine increased acetylcholine outflow in rat HC, which should have a beneficial effect on cognitive function [86]. Therefore, further studies are required to reveal the function of olanzapine in this respect. Consistent partially with these results, region‐specific alterations in schizophrenia model rats were found in the M1R mRNA and protein expressions. Thus, in agreement with data obtained in patients, significantly lower M1R mRNA expression was detected in the CTX of Wisket samples, which was not influenced by olanzapine treatment [83]. By contrast no significant changes in the PFC, but enhanced M1R mRNA expression in the hippocampal samples of Wisket rats were observed, which is controversial to human data [18, 83, 84]. Olanzapine treatment significantly enhanced both the mRNA and protein expression in the PFC, suggesting the beneficial effects of this treatment in this respect. While significant effect of olanzapine treatment in the M1R mRNA expression in the HC was observed, no changes in the protein expression was observed, which suggest that this phenomenon did not translate in the receptor number changes. This is in contrast with a study that found significantly increased the M1R mRNA expression in the HC after 12 weeks of olanzapine treatment investigated in healthy rats [28], which controversy might be due to the differences in experimental paradigm. However, the inconsistency between the clinical and preclinical findings probably due to the differences in the investigated area in human (dorsolateral part of PFC) vs. rat (total PFC), and/or the more complex structure of the human brain.

A limitation of this study is that only one dose of olanzapine was investigated, however, this dose was applied earlier [51], and we considered the 3R principle (replacement, reduction, and refinement). Furthermore, this is the first study about the behavioral effects of olanzapine depot treatment, therefore, we suppose that even with one dose relevant information was provided by the acute and chronic behavioral tests. The investigation in large home cages with environmental enrichment and with reduced human contact may enhance the reproducibility of the behavioral alterations, and provide more ecological circumstance for the animals [87], however, to determine the influence of these factors was not the aim of the present study. The manual data acquisition was a time‐consuming process in the HM; therefore, only 2–2 h of 4 days were statistically analyzed. We plan to develop image analysis by machine learning, which will lead to simpler data collection methods. Another disadvantage of this study is that only male animals were investigated. Although, schizophrenia has higher rates and more severe clinical course in males than in females [88], further studies are required for revealing the effects of olanzapine treatment in females, as well.

## 5 Conclusion

In summary, the triple‐hit Wisket model rats had impaired behavioral characteristics in both acute reward‐based tests and undisturbed circumstances investigated for prolonged periods, and altered M1R expression in CTX and HC, which were influenced by chronic olanzapine treatment. These data suggest that the chronic olanzapine depot treatment can restore only few negative signs in this complex schizophrenia‐like rat model.

## Conflicts of Interest

The authors declare no conflicts of interest.

## Data Availability

The data that support the findings of this study are available from the corresponding author upon reasonable request.
